# Postoperative Endophthalmitis after Combined Cataract Extraction and iStent Inject Implantation

**DOI:** 10.1155/2023/3132866

**Published:** 2023-05-04

**Authors:** Johnson Huang, Minh T. Nguyen, Mai Tsukikawa, Andrew Chen

**Affiliations:** Department of Ophthalmology, University of Washington, Seattle, WA, USA

## Abstract

*Purpose.* To report a case of postoperative endophthalmitis after combined cataract extraction and iStent inject implantation. *Observation.* A 70-year-old male with a nuclear sclerotic cataract and primary open-angle glaucoma underwent an uneventful phacoemulsification cataract extraction with implantation of an intraocular lens and an iStent inject trabecular bypass stent. The patient was prescribed a postoperative regimen of ofloxacin 0.3% and prednisolone acetate 1%, 1 drop four times a day each. On postoperative day five, he presented to the emergency room for eye pain and had 4+ mixed cells in the anterior chamber (AC) without hypopyon or vitritis on exam. Prednisolone 1% eye drops were increased from four times a day to every two hours while awake. Overnight, he developed worsening vision and severe eye pain. The next morning, he was found to have increased AC cells, vitritis, and intraretinal hemorrhages and was diagnosed with endophthalmitis. The patient underwent a vitreous tap and intravitreal injections of vancomycin (1 mg/0.1 mL) and amikacin (0.4 mg/0.1 mL). Cultures grew *Staphylococcus epidermidis*. Lab work-up revealed underlying neutropenia. Visual acuity eventually recovered to 20/20. *Conclusion and Importance.* This report highlights a case of endophthalmitis associated with placement of the iStent inject. The infection was well-controlled after administration of intravitreal antibiotics without removal of the iStent inject, and visual acuity eventually recovered to 20/20. Surgeons should be aware of endophthalmitis risk following combined iStent inject placement, and good recovery is possible without removal of the implant.

## 1. Introduction

Postoperative endophthalmitis is a rare but potentially devastating complication after cataract surgery. A recent report from the Intelligent Research in Sight Registry found that postoperative endophthalmitis occurred in 0.04% of 8,542,838 cataract surgeries performed in the United States between 2013 and 2017, and the risk of endophthalmitis was three times higher when cataract surgery is combined with glaucoma surgery (0.04% vs. 0.12%, *p* < 0.0001) [1]. In more recent years, the number of minimally invasive glaucoma surgery (MIGS) combined with cataract surgery has increased substantially with a 4-fold increase between 2012 and 2016 in a retrospective study on Medicare beneficiaries [2].

Results from prospective, randomized, multicenter trials for trabecular meshwork bypass stents did not report any endophthalmitis events [3–5]. There have only been small case series and case reports. Starr et al. reported 2 cases of endophthalmitis out of 2101 cases of cataract surgery with iStents; otherwise, the incidence rate and the management of endophthalmitis after cataract surgery with a trabecular bypass stent are not well-defined [6–8]. The iStent inject (Model G2-M-IS, Glaukos Corporation, Laguna Hills, CA, USA) is a titanium implantable shunt placed through the trabecular meshwork into Schlemm's canal that was introduced in 2020. In this manuscript, we report a case of acute *Staphylococcus epidermidis* endophthalmitis after combined cataract extraction and iStent inject placement that was successfully resolved with intravitreal antibiotics.

## 2. Case Report

A 70-year-old male patient with a history of primary open-angle glaucoma in both eyes and pseudophakia in the right eye underwent uneventful clear corneal phacoemulsification with insertion of a posterior chamber intraocular lens (IOL) and iStent inject placement in the left eye. Postoperatively, the patient was placed on a regimen of ofloxacin 0.3% and prednisolone acetate 1%. On postoperative day five, he presented to the emergency department with left eye redness and pain. His visual acuity (VA) was 20/20 without correction, and intraocular pressure (IOP) was 12 mmHg in the postoperative eye. Slit lamp examination showed mild conjunctival injection, 4+ mixed cells, and 1+ flare in the anterior chamber (AC) but no hypopyon and no vitritis. Fundus examination with clear view to the posterior pole revealed no retinal hemorrhages, whitening, or other evidence of endophthalmitis. Examination of the right eye was unremarkable. The differential diagnosis included uveitic flare, retained lens material, late toxic shock syndrome, or atypical presentation for endophthalmitis. Prednisolone acetate was increased to every two hours while awake, and he was instructed to come back early the next day.

**Figure 1:**
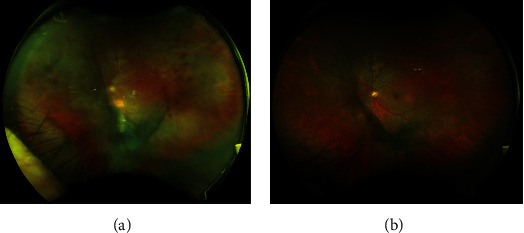
(a) Optos fundus photo of the left eye taken on postoperative day 4 shows diffuse vitreous haze, retinal hemorrhage, and whitening. (b) Optos fundus photo of the left eye taken on postoperative day 16 shows only vitreous debris.

The patient woke up the next morning with “a snow storm” in his vision and severe pain in his left eye. On examination, his VA dropped to 20/150, and he had increased AC cells, 4+ vitritis, and diffuse intraretinal hemorrhage, which was concerning for endophthalmitis (Figure 1(a)). He underwent an urgent vitreous tap and intravitreal injection of vancomycin 1.0 mg/0.1 mL and amikacin 0.4 mg/0.1 mL and was started on both oral moxifloxacin (400 mg daily) and moxifloxacin 0.5% eye drops four times a day. In the next day (postoperative day seven), the patient reported decreased pain. The exam demonstrated counting finger VA, a submillimeter hypopyon, and increased AC cell. The culture from his vitreous sample grew *S. epidermidis*, which was sensitive to both vancomycin and amikacin. Systemic bloodwork was only remarkable for stable and chronic pancytopenia attributed to long-standing cirrhosis. By postoperative day 16, his vision had improved to 20/20, with an exam only remarkable for vitreous debris (Figure 1(b)).

## 3. Discussion

In this report, we present a case of endophthalmitis that occurred after cataract extraction with iStent inject implantation. Despite many advancements in cataract surgery techniques, there remains a small but potential risk of serious postoperative endophthalmitis. Classic risk factors include older age, diabetes mellitus, posterior capsular tear, or wound leak [9]. The incidence of endophthalmitis after trabecular bypass stent placement is not well known with only a handful of cases having been published. However, endophthalmitis cases may increase as more cataract surgery combined with trabecular bypass stents are performed.

Endophthalmitis after cataract surgery performed in conjunction with trabecular meshwork bypass stents is rare. The randomized, prospective, multicenter trials for iStent, iStent inject, or Hydrus reported no endophthalmitis or other serious complications from combined stent implantation and cataract surgery [3, 4]. Chaves et al. reported endophthalmitis after a combined cataract extraction and first-generation iStent placement in an elderly patient who struck her eye while instilling drops two days after the surgery. This patient eventually developed no light perception despite a negative culture and two intravitreal injections of antibiotics [7]. Lam et al. reported a case of *Rothia mucilaginosa* endophthalmitis after phacoemulsification with insertion of two iStent injects, which progressed to retinal detachment requiring pars plana vitrectomy and removal of the IOL as well as iStents [8]. Starr et al. reported nine total cases of endophthalmitis associated with iStents on retrospective review of patients diagnosed with bacterial endophthalmitis after any MIGS procedure [6]. All cases received intravitreal injection of antibiotics, and none had the iStent removed. Four patients recovered their vision to 20/25 or better while the rest remained with the same level of decreased vision or lost their vision permanently. In these previously reported cases, there is a wide range of severity and visual outcomes.

The patient has a long-standing history of cirrhosis and pancytopenia. An immunocompromised state has been linked to endophthalmitis, but only for endogenous endophthalmitis such as in a previous report that described endogenous endophthalmitis in neutropenic patients [10]. To the best of our knowledge, no study has indicated an increased postoperative exogenous endophthalmitis risk in the setting of neutropenia. Alternatively, the patient's impaired immune response could account for the mild initial postoperative presentation of endophthalmitis with only anterior chamber cell and eye pain. Given the potential risk of complications related to intravitreal injection, a vitreous tap and injection of intravitreal antibiotics were deferred on initial presentation [11]. However, in this clinical scenario with the patient's baseline immunocompromised status, one could consider earlier vitreous tap and injection of antibiotics.

Though ceftazidime is the gold standard intravitreal antibiotic for gram-negative coverage, the decision was made to use amikacin due to a documented anaphylactic reaction to penicillin in the past. Recent studies suggest that the true cross-reactivity between penicillins and cephalosporins may be much more rare than previously thought, and authors have proposed that cephalosporins are safe to use even in those with a documented penicillin allergy [12, 13].

Cataract surgery with intracameral antibiotics has been shown to have a lower rate of endophthalmitis; however, it is not routinely used in the United States, in part due to the lack of approval by the Food and Drug Administration (FDA) [14]. Intracameral antibiotics were not available for our patient. It is unknown if intracameral antibiotics would confer additional protection against endophthalmitis for such cases.

We present a case where the patient recovered 20/20 vision without explantation in the setting of *S. epidermidis* endophthalmitis. For our case, explantation of the iStent was discussed due to the possible risk of the titanium implant serving as a nidus for biofilm formation and creating a reservoir for chronic infections [15]. There are no established guidelines for the removal of titanium intraocular implants. To date, the iStents that have been isolated have not been reported to be examined or individually cultured to determine if biofilm formation had occurred. One consideration for management could be the virulence of the organism, which would also play a role in the final visual outcome [15, 16]. Another consideration is the proclivity of bacterial species or strain to form biofilms. Starr et al. reported that no iStents were removed with a wide range of visual recovery. More studies would be required to better understand if early removal of the implants would be more beneficial for specific bacteria. Implant removal in MIGS-associated endophthalmitis may not be necessary in cases involving less virulent organisms.

## 4. Conclusions

With the rise of combined cataract extraction and MIGS, additional research is needed to determine the risk of endophthalmitis and guide the management of postoperative endophthalmitis involving implants. Our case demonstrates the clinical course of endophthalmitis after cataract surgery combined with iStent implantation from its early presentation to resolution with 20/20 visual acuity without explanting the stent. Prompt intervention and the more benign nature of *S. epidermidis* likely contributed to the favorable outcome [16]. This case suggests that iStent-associated endophthalmitis can be managed with conventional approaches without explanting the stent. Further studies are necessary to establish if and when explantation is necessary.

## Data Availability

All data generated or analyzed during this study are included in this article. Further enquiries can be directed to the corresponding author.
